# Dynamical nonequilibrium molecular dynamics reveals the structural basis for allostery and signal propagation in biomolecular systems

**DOI:** 10.1140/epjb/s10051-021-00157-0

**Published:** 2021-07-20

**Authors:** A. Sofia F. Oliveira, Giovanni Ciccotti, Shozeb Haider, Adrian J. Mulholland

**Affiliations:** 1 School of Chemistry, Centre for Computational Chemistry, University of Bristol, Bristol, BS8 1TS UK; 2 BrisSynBio, Life Sciences Building, Tyndall Avenue, Bristol, BS8 1TQ UK; 3 Institute for Applied Computing “Mauro Picone” (IAC), CNR, Via dei Taurini 19, 00185 Rome, Italy; 4 School of Physics, University College of Dublin, UCD-Belfield, Dublin 4, Ireland; 5 Università di Roma La Sapienza, Ple. A. Moro 5, 00185 Rome, Italy; 6 School of Pharmacy, University College London, London, WC1N 1AX UK

## Abstract

**Abstract:**

A dynamical approach to nonequilibrium molecular dynamics (D-NEMD), proposed in the 1970s by Ciccotti et al., is undergoing a renaissance and is having increasing impact in the study of biological macromolecules. This D-NEMD approach, combining MD simulations in stationary (in particular, equilibrium) and nonequilibrium conditions, allows for the determination of the time-dependent structural response of a system using the Kubo–Onsager relation. Besides providing a detailed picture of the system’s dynamic structural response to an external perturbation, this approach also has the advantage that the statistical significance of the response can be assessed. The D-NEMD approach has been used recently to identify a general mechanism of inter-domain signal propagation in nicotinic acetylcholine receptors, and allosteric effects in $\upbeta$-lactamase enzymes, for example. It complements equilibrium MD and is a very promising approach to identifying and analysing allosteric effects. Here, we review the D-NEMD approach and its application to biomolecular systems, including transporters, receptors, and enzymes.

**Graphic abstract:**

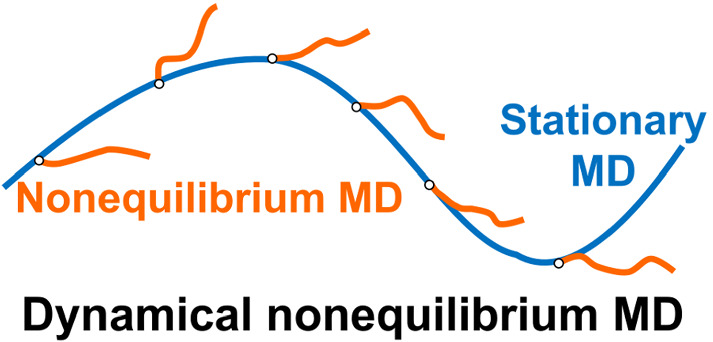

## Introduction

The binding of ions and small ligands to proteins, changes in voltage, pH, and temperature or the absorption of light by light-harvesting complexes are examples of external perturbations that can induce changes in protein structure and dynamics, thus affecting their function. Several approaches have been developed to study conformational changes in proteins *in silico* and understand the way they shape function (e.g., [1–3]). Some of these approaches rely on the use of information from equilibrium molecular dynamics (MD) simulations (e.g., [4–11]). However, for most conformational changes of interest in proteins, equilibrium MD simulations cannot access the relevant timescales and the number of events needed to determine the time-dependent structural changes and the order of the events associated with it, in a statistically significant way (for a detailed discussion about the ‘sampling problem’, see, e.g., [1, 12, 13]).

More than 4 decades ago, Ciccotti et al. [14] developed an approach that allows for the computing of the dynamic response of a system to an external perturbation. This dynamical approach to nonequilibrium molecular dynamics (D-NEMD) uses a combination of MD simulations in stationary (without any perturbation) and nonequilibrium (under the effect of external perturbations) conditions. In their work, Ciccotti et al. [14] developed a rigorous way to calculate averages for the observables of interest in nonequilibrium conditions under the effect of either instantaneous or time-dependent perturbations. Moreover, by directly comparing the stationary (e.g., equilibrium) and nonequilibrium simulations at the same point in time, the D-NEMD approach was used to reduce the statistical noise at short times and compute the time evolution of the dynamic response of a system to small perturbations [15–18]. When using the D-NEMD approach to determine the structural response of biomolecular systems (see next section for more details), the average difference at equivalent time points between trajectories in stationary (possibly equilibrium) and nonequilibrium settings is also used. However, this is done, because the observable of interest is the average divergence between the stationary and nonequilibrium trajectories due to the perturbation and not to reduce the noise as enough statistics can be gathered to compute the average nonequilibrium property and longer simulation times are being considered. In the past, the D-NEMD approach was mostly used to study the dynamics and the properties of fluids (e.g., [14, 15, 19–27]), but, surprisingly, it has been little used in recent years. In the biomolecular simulation field, only a few D-NEMD applications have been reported in the literature (e.g., [28, 29]) until very recently. We suggest that the reasons for this are twofold: first, the method was not well known in the biomolecular simulation community, which prevented it from being more widely used for biological questions; second, the sampling needed to achieve statistically significant responses for large, complex biomolecular systems was until recently out of range. Over the last decade, improvements in forcefields, algorithms, MD software, and computer hardware have allowed for longer simulation times and made it possible to run tens/hundreds/thousands of replicates in an acceptable timeframe [30]. Recent years have seen a resurgence in the use of the D-NEMD approach, with researchers from different fields realising how powerful, generally applicable and helpful the approach is, not only to study fluid dynamics (e.g., [31–33]) but also biological problems such as signal transmission in proteins and allostery (e.g., [34–37]).

Here, we briefly discuss the essential features of the D-NEMD approach and how it can be used in a more general setting to study proteins. We also review examples of studies that applied this approach to biological systems. The examples range from soluble enzymes to membrane proteins, and allow for the analysis of processes ranging from signal transmission to conversion of chemical energy into structural changes and identification of allosteric networks, as we discuss below.

## D-NEMD approach and the Kubo–Onsager relation

We will not go through a detailed and exhaustive description of the theoretical framework underlying Ciccotti et al.’s D-NEMD approach: for that, we invite the reader to consult references [16–18]. We will, however, briefly explain the rationale of the approach and illustrate how to compute the time-dependent macroscopic dynamic responses of a system by averaging over a (large) sample of nonequilibrium trajectories.

The rationale for the D-NEMD approach is simple. It can be summarised as follows: when an external perturbation is introduced into a system sampling a stationary (e.g., equilibrium) state, the system’s response to that perturbation can be directly obtained by comparing the reference and perturbed simulations at the same point in time. Therefore, let us consider ${\Gamma =}{(r}_{i}, \, p_{i}{)}$ as a point in the phase space for a dynamic system with N particles, where $r_{i}$ and $p_{i}$ are the coordinate–momentum pairs for the Hamiltonian dynamics. Additionally, let us also consider that the system’s Hamiltonian changes with time, $H({\Gamma ,}t{)}$. A macroscopic observable at a given time *t*, *O*(*t*), can be obtained as an ensemble average in phase space of the corresponding microscopic observable, $\hat{\text {O}}(t)$

1 $$\begin{aligned} O(t)\equiv \langle \hat{\hbox {O}}(\Gamma )w(\Gamma , t)\rangle ; \end{aligned}$$

$w{(\Gamma ,}t{)}$ is the phase-space probability density, and $\langle \rangle$ indicates the average over the phase space. From the Liouville equation, we have

2 $$\begin{aligned} w{(\Gamma ,}t{)=}S^{{\dag }}(t)w{(\Gamma ,0)}, \end{aligned}$$

where $S^{{\dag }}(t)$ is the adjoint of the time evolution operator of the dynamical system, *S*(*t*). As such, the microscopic observable $\hat{\text {O}}(\Gamma (t))$ can be defined as

3 $$\begin{aligned} \hat{\text {O}}(\Gamma (t))=S(t)\hat{\text {O}}(\Gamma (0)). \end{aligned}$$

By combining the previous equations, we obtain the Kubo–Onsager relation [14, 15]

4 $$\begin{aligned} O(t)\equiv & {} \langle \hat{\hbox {O}}(\Gamma )[S^{{\dag }}(t)w{(\Gamma ,0)}]\rangle \nonumber \\= & {} \langle [S(t){\hat{\text {O}}}(\Gamma )]w(\Gamma , 0)\rangle . \end{aligned}$$

Equation 4 explains that the ensemble average of the microscopic observable $\hat{\text {O}}(\Gamma )$ over the time-dependent probability density $w(\Gamma ,t)$ at a certain time *t* is the same as the ensemble average of the microscopic observable at the point $\Gamma (t)$, corresponding to the time evolution of the initial phase-space point $\Gamma$(0), averaged over the probability density at time 0, $w{(\Gamma ,}0)$. Thanks to the Kubo–Onsager relation, a nonequilibrium macroscopic average can be computed by the expected value of the time evolved observable over the initial ensemble. Note that $w(\Gamma ,0)$ needs to be a stationary state (e.g., an equilibrium or metastable state) in order for it to be adequately sampled using MD simulations. All nonequilibrium trajectories (along which the microscopic observable $\hat{\text {O}}(\Gamma (t))$ is computed) start from these stationary sampled points. The time-dependent behaviour of the macroscopic observable, *O*(*t*), can be estimated by averaging $\hat{\text {O}}(\Gamma (t))$ over all the nonequilibrium trajectories. Note that the statistical errors associated with *O*(*t*) can be computed to assess the significance of the results, and, if needed, they can be reduced further by simply running additional nonequilibrium trajectories.

**Fig. 1:**
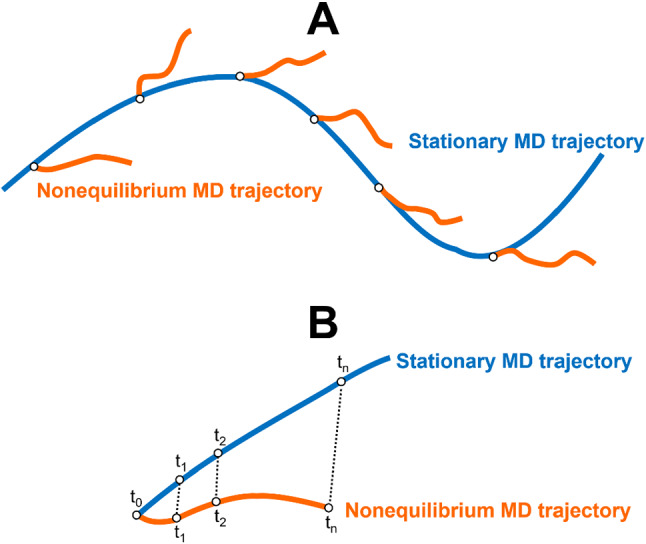
**A** Schematic representation of the D-NEMD approach. The stationary (e.g., equilibrium) MD trajectory (blue line) provides the initial distribution for the nonequilibrium simulations at time t$=$0 (orange lines). The individual nonequilibrium trajectories sample the dynamic evolution of the system after the introduction of a perturbation. **B** Scheme for extracting the structural response of a protein to a perturbation. For each pair of stationary and nonequilibrium trajectories, the system’s response to the perturbation is obtained by comparing the position of each individual C$\upalpha$ atom in both trajectories at equivalent points in time. The statistical average of the response as a function of the time is then determined, and the corresponding statistical errors calculated

In practice, the procedure to set up the nonequilibrium simulations is straightforward (Fig. 1A) and consists of, first, running long MD simulations for the stationary (e.g., equilibrium) reference state to generate a distribution of configurations for the system of interest (unperturbed simulations). These conformations are then used as the starting points for an ensemble of nonequilibrium trajectories under the effect of the external perturbation (perturbed simulations). The nonequilibrium trajectories are then integrated until time *t*.

The nature of the perturbation to be introduced in the system will depend on whatever question the user wants to address, and it can be either instantaneous or gradual. A very diverse range of perturbations has already been reported in the literature, ranging from the introduction of external electric fields and thermal gradients (e.g., [15, 22, 24, 33]) to the switching off of radiation flows [29], ATP hydrolysis [28, 35], and the removal of ligands from their binding sites [34, 36, 37]. The response of the system can be extracted using the Kubo–Onsager relation (Eq. 4) through the direct comparison of the observable of interest between each pair of unperturbed stationary and “branching” perturbed nonequilibrium trajectories at equivalent time *t*, followed by the averaging of the difference over all pairs of simulations. Note how easy and direct it is with this approach to measure the response of the system as it simply requires the subtraction of the observable between two trajectories. In particular, for the structural response of proteins (Fig. 1B), the difference in the position of each individual C$\upalpha$ atom between pairs of stationary and nonequilibrium trajectories at a given time point can be determined and afterwards averaged over all pairs of simulations. The time evolution of the average difference in position will highlight the cascade of events associated with protein’s structural response. The use of the C$\upalpha$ atoms for this analysis is a straightforward way to identify the most pronounced conformational rearrangements. Besides, there is also the practical advantage that C$\upalpha$ motions are less subject to noise and as such, converge more rapidly than, e.g., sidechains.

The use of D-NEMD to study the structural response of proteins allows for not only the identification of the conformational changes, but also the mapping of the time evolution of such rearrangements. This approach has advantages when compared to standard equilibrium MD simulations: extracting the conformational response of the protein is straightforward and, as explained above, can be done easily by directly comparing each pair of unperturbed equilibrium and perturbed nonequilibrium trajectories at equivalent times; the statistical significance of the response can be assessed and, if needed, the statistical error associated with the response can be made as small as necessary just by increasing the number of nonequilibrium trajectories. However, despite the advantages stated above, it is important to bear in mind that in D-NEMD, the perturbation(s) introduced in the system depends on the question and user’s choice, and that different perturbations may give different results and might identify different communication networks. As such, careful thought needs to be given to the choice of perturbation, and it is up to the user to decide which is the most suitable and relevant for a given biological question. This is not always obvious and, in some cases, figuring out which is the most (biologically) relevant trigger(s) behind functionally relevant conformational changes is not an easy task (for an example of this, see the “ATP-binding cassette transporters” section below). Our only advice here is for the users to be familiar with the details of the system to be studied and have a clear idea of the question they want to answer. Another important point that should be highlighted is that it is not possible to *a priori* know the amount of sampling needed to obtain statistically significant structural responses, as this will depend not only on the system but also on the perturbation introduced. Determining the statistical errors associated with the structural response is important to test if the sampling gathered is sufficient.

In the following section, we discuss some applications of the D-NEMD approach to proteins, demonstrating the versatility of the approach for a variety of complex biomolecular problems. Note that in the examples below, all (equilibrium and nonequilibrium) simulations were performed using standard biomolecular simulation software, namely GROMACS [38–40] and AMBER [41].

## ATP-binding cassette transporters

**Fig. 2:**
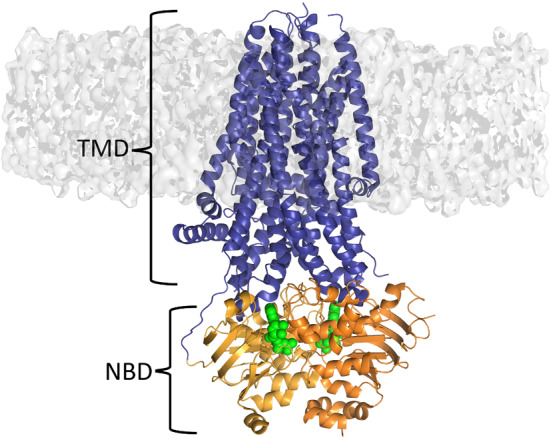
Basic architecture of an ABC transporter. Regardless of the direction of the transport, all ABC transporters are composed of a minimum “functional core” formed by two catalytic domains (NBD) and two transmembrane domains (TMDs). The NBDs, TMDs, and ATP molecules are coloured in orange, blue, and green, respectively. This figure represents the cryo-EM structure of the phosphorylated cystic fibrosis transmembrane conductance regulator (CFTR) channel (PDB code: 6O1V) [46]

ATP-binding cassette (ABC) transporters are ubiquitous to all kingdoms of life and form one of the most prominent families of integral membrane proteins [42, 43]. This family includes not only importers and exporters but also channels and receptors [44]. ABC transporters translocate a variety of substrates across the membrane [42, 43], ranging from ions to lipids, drugs, toxins, and peptides. For that, the proteins harness the free energy of ATP binding and hydrolysis to drive the transport of the substrates. The human genome encodes for approximately 50 ABC transporters involved in a plethora of cellular processes [42, 43], e.g., cholesterol, fatty acid and lipid transport, chloride homeostasis, and multidrug resistance. Mutations and malfunction of this family of proteins have been associated with several diseases, including cystic fibrosis, Tangier and Stargardt diseases, and hypercholesterolaemia [45]. The basic architecture of ABC transporters comprises two nucleotide-binding domains (NBDs) that provide the engine that drives the transport and two transmembrane domains (TMDs) that are embedded in the membrane and form the translocation pathway (Fig. 2).

While significant advances have been achieved in understanding ABC transporters’ working mechanisms (e.g., [47]), how the energy of ATP hydrolysis is harnessed and converted into a wave of conformational changes that propagate from the ATP-binding sites to the translocation pathways is still unclear. About 10 years ago, Damas et al. [28] used the D-NEMD approach and the Kubo–Onsager relation (Eq. 4) to study the conformational events that occur as a response to ATP hydrolysis in the hemolysin B (HlyB) NBD dimer and understand how those structural changes propagate within the catalytic domains. This work was one of the first applications of the D-NEMD approach to biomolecular systems. HlyB is part of the hemolysin A type I secretion system in *Escherichia coli *and it transports the unfolded exotoxin hemolysin A [48].

**Fig. 3:**
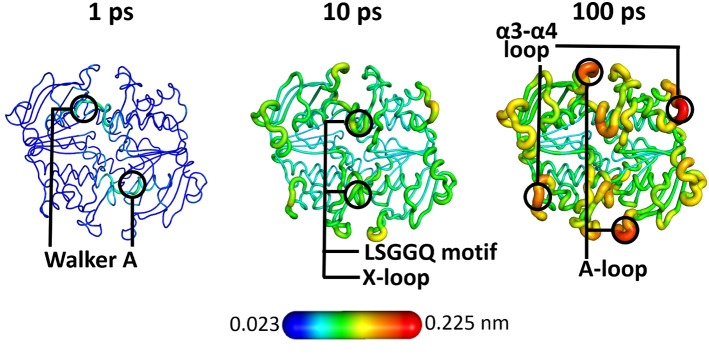
Structural response of the HlyB NBD dimer to ATP hydrolysis. The structural deviation of the protein’s C$\upalpha$ atoms at specific times (1, 10, and 100 ps) after ATP hydrolysis is mapped on the average perturbed HlyB structure according to the scale. Cartoon thickness is also related to the conformational response. This figure was adapted from reference [28]

Damas et al. [28] performed 100 very short (100 ps long) nonequilibrium simulations in which the ATP molecules bound to the ATP-binding sites were converted into ADP and inorganic phosphate (Pi), thus mimicking hydrolysis. It is important to emphasise here that the nature of the perturbation introduced depends on the question the user wants to address and the system to be studied. Identifying the most relevant trigger(s) behind the conformational rearrangements in ABC transporters is not easy: is it the change in electrostatic potential within the binding site upon hydrolysis, the release of inorganic phosphate, or the combination of the previous two. As such, choosing the adequate perturbation for D-NEMD is far from straightforward. Since Damas et al. were interested in identifying how the energy from ATP hydrolysis is converted into a wave of conformational changes, they used as a perturbation the conversion of ATP into its hydrolysis products [28], which is a sensible and reasonable choice for the question they were addressing. The protein’s structural response was then extracted using the Kubo–Onsager relation (Eq. 4) and comparing the ATP-bound equilibrium simulations with the ADP.Pi-bound nonequilibrium simulations in the 100 ps after hydrolysis [28]. These simulations showed how HlyB converts the energy of ATP hydrolysis into mechanical work by inducing conformational changes in specific regions of the protein. The simulations also allowed for the identification of the first steps associated with the propagation of the structural rearrangements within the HlyB NBDs [28]. Damas et al. [28] were able to show that immediately after ATP hydrolysis, the motifs forming the ATP-binding sites, notably Walker A and LSGGQ motif, undergo a conformational rearrangement which is afterwards transmitted progressively to other regions of the dimer, particularly the X loop, A loop, and $\upalpha 3-\upalpha 4$ loop (Fig. 3). The fact that the X loops respond very quickly to ATP hydrolysis is fascinating as these motifs are in direct contact with the TMDs coupling helices. We can then speculate that any structural rearrangements occurring in the X loops can swiftly be propagated to the TMDs and transmitted to the translocation pathway.

**Fig. 4:**
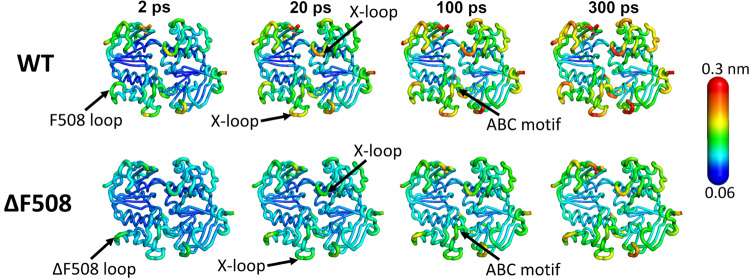
Differences in the structural response to ATP hydrolysis between the wild-type and $\Delta$F508 mutant NBD dimer of CFTR from D-NEMD simulations. The structural deviation of the dimer’s C$\upalpha$ atoms at specific times (2, 20, 100, and 300 ps) after ATP hydrolysis are mapped on the average perturbed post-hydrolysis structures according to the scale. This figure was adapted from reference [35]

Recently, Abreu et al. [35] also used the D-NEMD approach to understand the impact of a mutation on signal propagation within the NBD dimer of the cystic fibrosis transmembrane conductance regulator (CFTR) channel. The CFTR protein is an ion channel that belongs to the ABC transporter family [49] and is responsible for chloride and bicarbonate homeostasis [49]. Mutations in this channel can cause cystic fibrosis, one of the most common lethal genetic diseases in Caucasian populations [50, 51]. Although thousands of mutations that interfere with CFTR synthesis, processing, and function have been reported, the deletion of a phenylalanine residue in position 508 ($\Delta$F508) is the most common cystic fibrosis-causing mutation [50]. In their work, Abreu et al. [35] performed 460 short (300 ps long) nonequilibrium MD simulations for the wild-type and $\Delta$F508 NBDs in order not only to characterise the protein’s response to ATP hydrolysis in a time-dependent manner but also to understand how the mutation affects signal transmission within the NBD dimer [35]. In this case, the perturbation introduced in the system was similar to the one used by Damas et al. [28], notably the conversion of ATP into ADP and Pi. Overall, the comparison between the wild-type and $\Delta$F508 mutant responses clearly shows that the same key functional motifs are involved in harnessing ATP hydrolysis’ energy. However, the mutant is less dynamic, and a slower propagation of the conformational changes is observed compared to the wild-type (Fig. 4). Note that, e.g., 300 ps after hydrolysis, the mutant displays higher rigidity than the wild-type, showing smaller structural changes throughout the entire NBDs, including in key functional motifs such as Walker A, X loops, and ABC motifs. The $\Delta$F508 mutation delays the transmission of energy (after hydrolysis) from the ATP-binding site to the rest of the NBDs and likely to the TMDs, thus ultimately impacting the ion channel’s opening and closing.

The comparisons of the cryo-EM structures of the ATP-bound and orthovanadate-trapped (which mimics the hydrolysis-transition state) of the *Thermus thermophilus* multidrug-resistance proteins A and B from Hofmann et al. [52] show that ATP hydrolysis induces small changes in the specific regions of the NBDs. The differences between the experimental structures of TmrAB in the pre-hydrolysis and hydrolysis-transition state qualitatively agree with the conformational rearrangements reported by Damas et al. [28] and Abreu et al. [35] when using the D-NEMD approach. For instance, the experimental structures show subtle conformational differences in the regions surrounding the canonical ATP-binding site, notably Walker A and A loop of monomer A and ABC motif and X loop of monomer B. Some of these regions were identified by the D-NEMD approach to respond to ATP hydrolysis in both the HlyB [28] and CFTR [35] NBD dimers, e.g., X loop and ABC motif.

## Nicotinic acetylcholine receptors

**Fig. 5:**
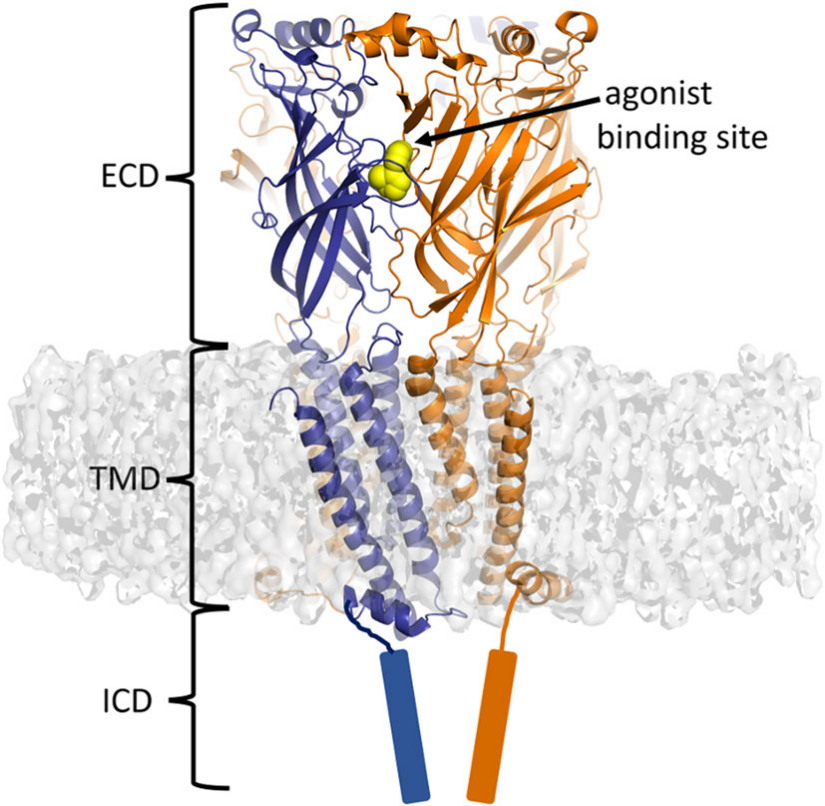
Representation of the general architecture of a nicotinic acetylcholine receptor (nAChR). The X-ray crystallographic structure of $\upalpha 4\upbeta 2$ nAChR (PDB code: 5KXI [63]) is used for illustrating the three distinct domains forming nAChR receptors: the extracellular domain (ECD), the transmembrane domain (TMD), and the intracellular domain (ICD). The principal and complementary subunits are coloured in blue and orange, respectively, and nicotine (which is bound to the agonist binding site) is represented in yellow spheres. Note that the ICDs are not present in the 5KXI X-ray structure and were added to this image only as a reference

Nicotinic acetylcholine receptors (nAChRs) are cation-selective ion channels that belong to the superfamily of pentameric ligand-gated ion channels [53–55]. In humans, this superfamily besides nAChRs also includes several other important cation and anion-permeable channels, such as the serotonin type 3 (5- $\hbox {HT}_{{3}})$ receptor, $\upgamma$-aminobutyric acid type A ($\hbox {GABA}_{\mathrm {A}})$ receptor, glycine receptor, and the zinc-activated ion channel (ZAC) [53, 54, 56–58]. nAChRs are expressed throughout the central nervous system and at the neuromuscular junction and autonomic ganglia, where they contribute to various functions, including cognition and reward [59, 60]. These receptors are putative targets for the treatment of a variety of neurodegenerative diseases, neurodevelopmental disorders, pain, and (nicotine) addiction [61]. All nAChRs are formed by the homo- or hetero-assembly of five subunits arranged in an approximately symmetric manner around a central cation-conducting channel [58, 61]. The individual subunits share a similar structure, formed by a large N-terminal extracellular domain (ECD) where the agonist-binding site is located, a transmembrane domain (TMD) that surrounds the ion-conducting pore and an intracellular domain (ICD) of variable length and structure [53–55] (Fig. 5). The binding of an agonist, such as acetylcholine or nicotine, to the orthosteric site triggers the opening of the ion channel (gating), allowing for positively charged ions ($\hbox {Na}^{{+}}$ and $\hbox {Ca}^{{2+}})$ to passively diffuse across the membrane, triggering depolarisation and signalling mechanisms [54, 62].

**Fig. 6:**
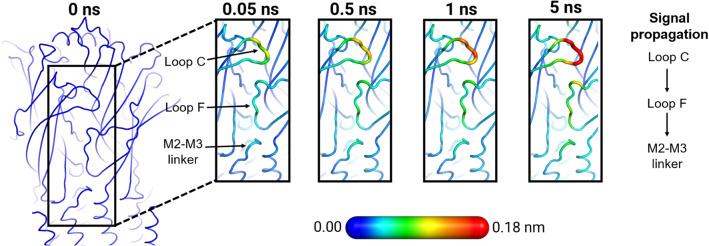
Signal propagation pathway from the ECD to the TMD in the human ${{\upalpha }}$4${{\upbeta }}$2 nAChR from D-NEMD simulations. Average C${{\upalpha }}$-positional deviation at times 0, 0.05, 0.5, 1, and 5 ns following nicotine annihilation. The structural response of the receptor was extracted using the Kubo–Onsager relation (Eq. 4). The time evolution of the response of the receptor is mapped onto the average structure for the system without nicotine using the colour scheme in the scale. This image was adapted from reference [37]

While the application of a variety of experimental and computational approaches together with the experimental structures available (e.g., [63–65, 67]) have led to a greater insight into the function of nAChR, the structural changes induced by agonist binding/unbinding and how those changes are propagated to the ion channel remain poorly understood. Answering this question requires an approach that allows us to follow the receptor’s time-dependent response as its conformation adapts to the agonist binding or unbinding. Recently, we used the D-NEMD approach to identify the structural features involved in signal propagation upon nicotine removal in the human $\upalpha 4\upbeta 2$ nAChR [37]. Extensive $\mu$s-long equilibrium simulations were used as the starting point for hundreds (>400) short nonequilibrium simulations, in which nicotine was removed from the orthosteric ligand-binding pocket, thus forcing signal transmission via the reaction of the receptor and showing the mechanical and dynamic coupling between structural elements involved in such response [37]. These simulations revealed a striking pattern of communication between the binding pockets and the transmembrane domains and showed the sequence of conformational changes associated with the initial steps of inter-domain signal propagation (Fig. 6).

**Fig. 7:**
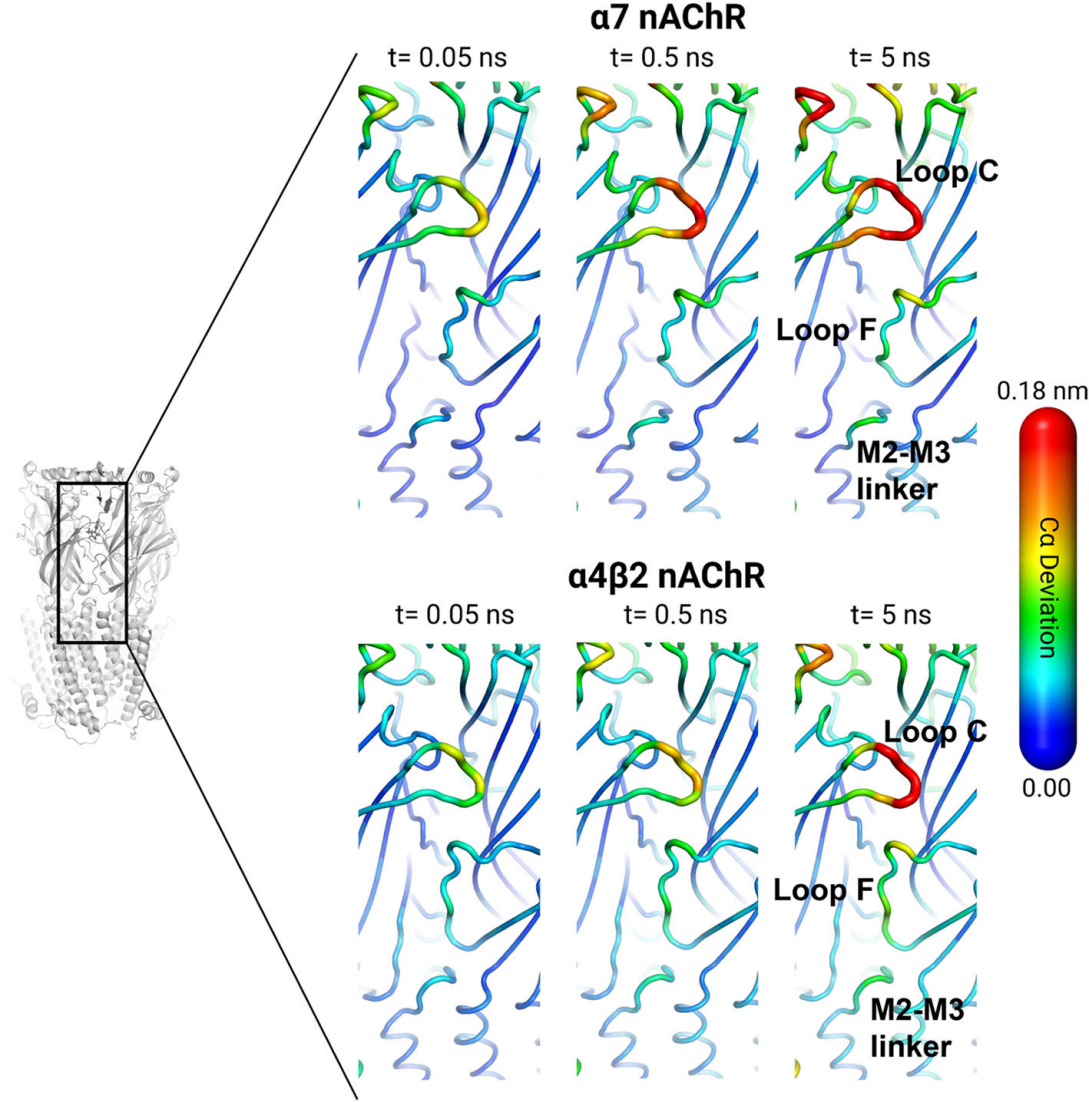
General mechanism for signal transduction in nAChRs revealed by D-NEMD simulations. Comparison between inter-domain signal propagation pathways in the human $\upalpha 7$ and human $\upalpha 4\upbeta 2$ nAChRs. Note that although there are differences in the apparent rates of propagation between the two receptors, the sequence of conformational changes associated with the initial steps of signal transmission is the same, i.e., the structural elements involved are the same. This image was adapted from references [37] and [34]

We have also used the D-NEMD approach and the Kubo–Onsager relation (Eq. 4) to study inter-domain communication in the human $\upalpha 7$ nAChR [34]. The comparison of the responses between the two different nAChR subtypes allowed for the identification of a general mechanism for inter-domain communication within this family, with the structural motifs involved in ECD/TMD signal transmission and the sequence of structural changes being highly conserved across the family [34, 37]. Signal propagation from the agonist-binding site to the TMDs starts with the structural rearrangements in loop C, which are then propagated to loop F and finally to the TMDs via the M2–M3 linker (Fig. 7). The nonequilibrium simulations also showed differences in the rate of propagation of the structural changes, which may relate to differences in function and response between receptor subtypes [34].

## Class A $\upbeta$-lactamases

$\upbeta$-lactamases are bacterial enzymes capable of hydrolysing $\upbeta$-lactam antibiotics [68]. The expression of these enzymes is the primary mechanism of antimicrobial resistance in Gram-negative bacteria [68]. $\upbeta$-lactam antibiotics, e.g., penicillin, function by inhibiting bacterial penicillin-binding proteins, a group of enzymes that catalyse transpeptidation and transglycosylation reactions during cell wall biosynthesis [68]. $\upbeta$-lactamases hydrolyse the amide bond of the four-membered $\upbeta$-lactam ring in these antibiotics, resulting in a product that does not inhibit penicillin-binding proteins [69], thus conferring resistance. Antimicrobial resistance is a global public health issue, with many antimicrobial compounds becoming ineffective against previously susceptible microorganisms [68].  $\upbeta$-lactamases are divided into classes A, B, C, and D [68], depending on whether they are serine hydrolases (classes A, C, and D) or metalloenzymes (class B). Class A enzymes, in particular, are the most widely distributed and studied $\upbeta$-lactamases, and this class includes the SHV, CTX-M, TEM, and KPC enzymes [68].

**Fig. 8:**
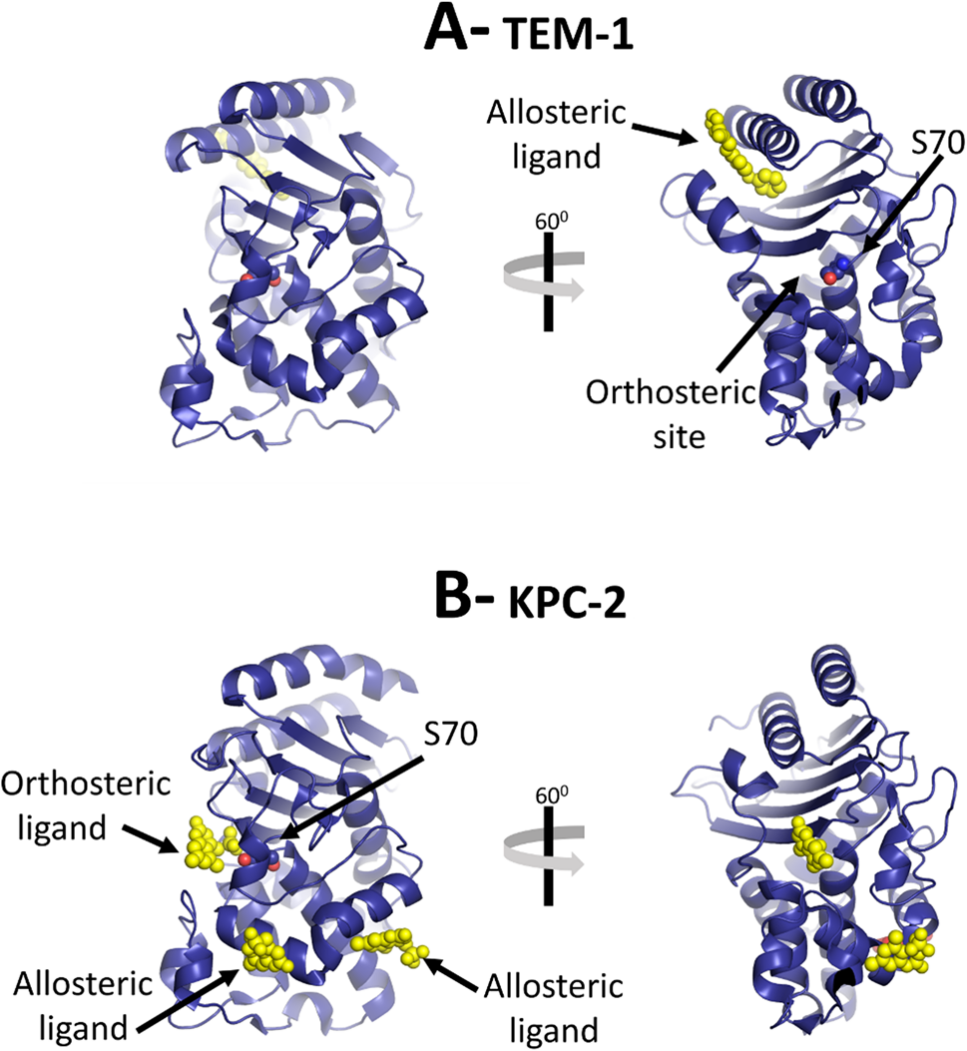
Structures of two class A $\upbeta$-lactamases. **A** X-ray structure of TEM-1 enzyme (PDB code:1PZP) [75]. **B** X-ray structure of KPC-2 enzyme (PDB code:6D18) [71]. The yellow spheres represent orthosteric and allosteric ligands bound to the enzymes (orthosteric ligands bind at the active site; allosteric ligands bind at other sites, distant from the active site)

Although the mechanism of covalent inhibition is well established in class A $\upbeta$-lactamases [70], the use of allosteric sites as an inhibition strategy is much less well explored. Furthermore, in the cases where allosteric inhibition has been achieved (e.g., [71, 72]), the mechanism by which it happens is poorly understood. Allosteric sites are distinct and spatially distant from the (orthosteric, catalytic) active site (Fig. 8). Molecules binding at allosteric sites may modulate (e.g., inhibit) the activity of the enzyme (e.g., [71, 72]), by means that are generally not well understood. With this in mind, Galdadas et al. [36] used the D-NEMD approach to examine effects of allosteric ligands for two clinically relevant class A $\upbeta$-lactamases, namely the TEM-1 and KPC-2 enzymes (Fig. 8). The results showed that changes at an allosteric site were transmitted to the active site, and identified the pathways that connect them. TEM-1 is one of the most common $\upbeta$-lactamase in Gram-negative bacteria, and its hydrolytic activity is limited to penicillins and early generation cephalosporins [69]. Nevertheless, mutations have allowed for subsequent variants to hydrolyse broad-spectrum cephalosporins [73]. KPC-2 (*Klebsiella pneumoniae* carbapenemase-2) is a highly versatile $\upbeta$-lactamase with a broad spectrum of substrates that includes penicillins, cephamycins, and carbapenems [74]. Understanding the conformational rearrangements taking place upon ligand (un)binding to the allosteric sites of TEM-1 and KPC-2, and how such changes are communicated to the active site, may provide a new strategy for the rational development of novel allosteric inhibitors.

**Fig. 9:**
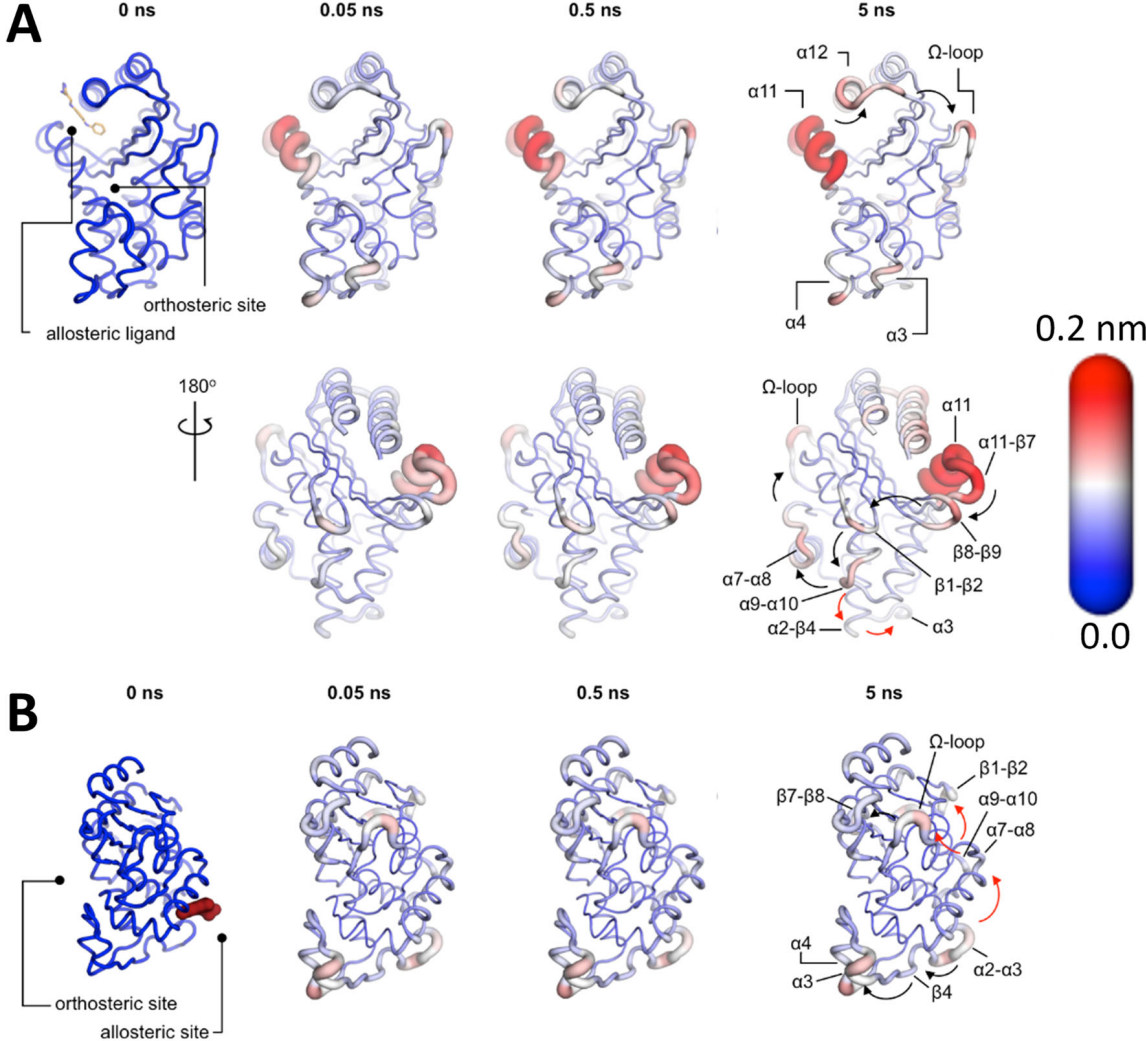
D-NEMD simulation pathways reveal communication pathways in TEM-1 (**A**) and KPC-2 (**B**) class A $\upbeta$-lactamase enzymes, showing how changes in binding at an allosteric site (distant from the active site) are transmitted to the enzyme active site. The amplitude of the C$\upalpha$ structural response to the removal of an allosteric ligand is coloured according to the scale on the right. The cartoon thickness is also related to the amplitude of the conformational response. The arrows mark the direction of the propagation of the signal upon the removal of the allosteric ligand. The red and the black arrows highlight different routes for the signal to propagate. This image was adapted from reference [36]

Galdadas et al. [36] performed 800 short nonequilibrium simulations (400 for each enzyme), and in each simulation, the allosteric ligand was removed from its binding site. The response of the system was extracted using the Kubo–Onsager relation (Eq. 4), as in the examples above. In both enzymes, the cascade of conformational changes in response to the removal of an allosteric ligand revealed the pathways by which the structural changes are transmitted from the allosteric site to the enzyme’s catalytic active site [36]. In TEM-1 (Fig. 9A), the structural response starts at the allosteric site (between $\upalpha$11 and $\upalpha$12 helices) and proceeds via the $\upbeta$1-$\upbeta$2 loop to the $\upalpha$9-$\upalpha$10 loop. From there, the conformational changes bifurcate towards the $\Omega$ loop via the $\upalpha$7-$\upalpha$8 loop or towards the $\upalpha$3-$\upalpha$4 pivot via the $\upalpha$2-$\upbeta$4 loop. In KPC-2 (Fig. 9B), the structural response starting at the allosteric site (between $\upalpha$2 and $\upalpha$7 helices) propagates to loop $\upalpha$2-$\upbeta$4 and the $\upalpha$3-turn-$\upalpha$4 helix, and is then transmitted to the $\Omega$ loop via the $\upalpha$7-$\upalpha$8 loops. Additionally, the structural signal can also take another route from the $\upalpha$7–$\upalpha$8 loop towards the $\upbeta$9-$\upalpha$12 loop, which lies adjacent to the hinge region. Particularly striking is the fact that, in both TEM-1 and KPC-2, the allosteric signal starting from different sites on the protein converges on the structural elements that are involved in catalysis. Furthermore, more than 50% of the clinically relevant mutations can be mapped onto the above-described allosteric pathways [36]. Amino acid variations along these pathways are likely to impact signal transmission and modulate the conformational response of functional motifs in both enzymes. A detailed molecular description of the impact of mutations within the allosteric networks in TEM-1 and KPC-2 may help understand the relationship between sequence, dynamics, allosteric behaviour, and activity spectrum. The D-NEMD approach combining equilibrium and nonequilibrium simulations can help in revealing and analysing such allosteric behaviour.

## Perspective and prospects

Here, we have reviewed the D-NEMD approach developed by Ciccotti et al., to extract the time-dependent structural response of proteins to a perturbation. This approach can be used to address complex biological questions, such as allostery or intra-protein communication. It provides a rigorous way to study complex time-dependent phenomena using fundamental statistical mechanics. Although the appropriate perturbation introduced in the system depends on the biological question to be explored, extracting the time-dependent response is straightforward using the Kubo–Onsager relation. In the D-NEMD approach, the response of the system to the perturbation is determined by directly extracting the average time-dependent conformational changes. This approach also has the advantage that the statistical significance of the response can be assessed, and the associated errors computed and (and given sufficient computer) made as small as desirable by simply increasing the number of nonequilibrium trajectories. Of course, in some cases, there could also be the need to expand the initial distribution of conformations, i.e., to extend the unperturbed simulations further. Over the past decade, advances in computing power and software have made it feasible to carry out the long equilibrium simulations, and tens to hundreds of nonequilibrium simulations, necessary to obtain statistically significant responses. The examples given above, covering different proteins and a range of biological questions, demonstrate the versatility and general applicability of the D-NEMD approach. This approach can provide a comprehensive and unbiased mapping of the structural responses and communication networks in proteins. D-NEMD simulations complement standard equilibrium MD simulations. This method has great potential to be useful in revealing allosteric effects, identifying communication pathways and effects of mutation on these. The D-NEMD method promises new and detailed insights into the structural changes associated with signal transduction, enzyme catalytic cycles, and other biomolecular processes. The insights from D-NEMD simulations should help in understanding and predicting allosteric effects, thus facilitating the design and development of allosteric ligands (e.g., allosteric enzyme inhibitors).

## Data Availability

This manuscript has no associated data or the data will not be deposited. [Authors’ comment: Since this paper is a review, no data was generated. All relevant data is deposited as outlined in the original papers reviewed here.]
